# Operator Radiation and the Efficacy of Ceiling-Suspended Lead Screen Shielding during Coronary Angiography: An Anthropomorphic Phantom Study Using Real-Time Dosimeters

**DOI:** 10.1038/srep42077

**Published:** 2017-02-07

**Authors:** Qianjun Jia, Ziman Chen, Xianxian Jiang, Zhenjun Zhao, Meiping Huang, Jiahua Li, Jian Zhuang, Xiaoqing Liu, Tianyu Hu, Wensheng Liang

**Affiliations:** 1 Department of Cardiac Catheterization Lab, Guangdong Cardiovascular institute, Guangdong Provincial Key Laboratory of South China Structural Heart Disease, Guangdong General Hospital, Guangdong Academy of Medical Sciences, 96 Dongchuan Road, Guangzhou 510100, China; 2 Image Guided Therapy Systems, Philips Healthcare Australia, 747 Lytton Rd, Murarrie QLD 4172, Australia; 3 Department of Radiology, Guangdong General Hospital, Guangdong Academy of Medical Sciences, 96 Dongchuan Road, Guangzhou 510100, China; 4 Department of Cardiac Surgery, Guangdong Cardiovascular Institute, Guangdong Provincial Key Laboratory of South China Structural Heart Disease Guangdong General Hospital, Guangdong Academy of Medical Science, 96 Dongchuan Road, Guangzhou 510100, China; 5 Epidemiology Division, Guangdong Cardiovascular Institute, Guangdong Provincial Key Laboratory of South China Structural Heart Disease, Guangdong General Hospital, Guangdong Academy of Medical Sciences, Guangzhou, Guangdong, China, 96 Dongchuan Road, Guangzhou 510100, China

## Abstract

Operator radiation and the radiation protection efficacy of a ceiling-suspended lead screen were assessed during coronary angiography (CA) in a catheterization laboratory. An anthropomorphic phantom was placed under the X-ray beam to simulate patient attenuation in eight CA projections. Using real-time dosimeters, radiation dose rates were measured on models mimicking a primary operator (PO) and an assistant. Subsequently, a ceiling-suspended lead screen was placed in three commonly used positions to compare the radiation protection efficacy. The radiation exposure to the PO was 2.3 to 227.9 (mean: 67.2 ± 49.0) μSv/min, with the left anterior oblique (LAO) 45°/cranial 25° and cranial 25° projections causing the highest and the lowest dose rates, respectively. The assistant experienced significantly less radiation overall (mean: 20.1 ± 19.6 μSv/min, P < 0.003), with the right anterior oblique (RAO) 30° and cranial 25° projections resulting in the highest and lowest exposure levels, respectively. Combined with table-side shielding, the ceiling-suspended lead screen reduced the radiation to the PO by 76.8%, 81.9% and 93.5% when placed close to the patient phantom, at the left side and close to the PO, respectively, and reduced the radiation to the assistant by 70.3%, 76.7% and 90.0%, respectively. When placed close to the PO, a ceiling-suspended lead screen provides substantial radiation protection during CA.

Coronary angiography (CA, which uses ionizing radiation, irradiating the patient and the operating staff) is routinely performed to detect coronary artery stenosis and provide lesion visualization during percutaneous coronary interventions (PCIs)^1^,^2^. Radiation exposure in interventional cardiology is of the utmost importance because low but frequent doses of ionizing radiation can cause skin injuries, premature cataract development, and increase the lifetime risk of cancer^3^,^4^. Although CA is an established imaging technique, the ever-increasing workload in catheterization laboratories that use ionizing radiation raises concerns regarding the radiation safety of interventional cardiologists^5^,^6^.

Compared with fluoroscopy, CA uses higher X-ray energy to obtain a series of cineradiograms from various projection angles for a thorough examination of the coronary arteries. The exposure rate of operator radiation during CA might be more than tenfold that of fluoroscopy^7^. In addition, radiation level is correlated with the angiographic projection angle^8^, such that steep oblique projections cause greater amount of scatter^7^. When considering radiation protection, it is beneficial^9^ to raise operator awareness regarding scatter radiation variation when conducting interventional cardiac procedures.

Radiation shields play a vital role in protecting operators against scatter radiation in catheterization laboratories^9^,^10^,^11^,^12^. These shields should be used during procedures of any significant length^13^. Koukorava *et al*.^14^ and Shortt *et al*.^15^ reduced operator radiation up to 98% and 64% with the appropriate use of a ceiling-suspended shield and a lower body shield, respectively. However, the optimal placement of the ceiling-suspended shield remains unknown. Although Fetterly *et al*.^16^ suggested that maximum protection can be achieved during anterior posterior projection by placing the ceiling-suspended shield away from the radiation source, Koukorava *et al*.^17^ recommended that the shield be positioned close to the patient to create a “radiation shadow”.

Several investigations regarding operator radiation dose during CA have reported radiation dose measurements at various sensitive organs (e.g., the eye lens and the thyroid)^14^,^18^,^19^,^20^. In addition, previous work has been conducted to determine the influence of the angiographic projection angle on operator radiation^21^. Regardless of the reported data, a comprehensive understanding is lacking regarding operator radiation dose when combined with varying CA projection angles and shielding strategies. Furthermore, few published operator radiation studies have examined assisting operators who often work side-by-side with the primary operator (PO) during CAs and PCIs in training and complex procedures.

Conventionally, thermoluminescence dosimeters (TLDs) are worn by medical staff and retrospectively analysed over a period of time. Although widely applied in occupational dosimetry, this method cannot provide instantaneous feedback. Recent studies have recommended a new dosimeter (DoseAware, Philips Medical Systems, the Netherlands) that produces an operator radiation dose reduction by displaying real-time personal radiation levels in an cardiac catheterization lab^12^,^21^,^22^.

We sought to assess the operator radiation of the primary and assisting operators during CA at routine projection angles using real-time dosimeters. The shielding efficacy of a ceiling-suspended lead screen was tested in various positions to determine the optimal placement.

## Materials and Methods

### Experimental protocol in a cardiac catheterization lab

All the methods in current study were carried out in accordance with the approved guidelines. All experimental protocols in this retrospective study were approved by the Research Ethic Committee of Guangdong General Hospital. Informed consent was not obtained, because the subjects of this study were phantoms. Measurements were performed at the Guangdong General Hospital in a clinical cardiac catheterization laboratory using a Philips Allura Xper FD10 system (Philips Medical Systems, Best, the Netherlands). The clinical cineradiography setting was selected for acquisitions at 15 frames per second, with a 70–90 kV tube voltage and a 550–900 mA tube current under automatic exposure control and 0.1 mm Cu ±1.0 mm Al filtration with a 10-inch full field of view. Eight routine diagnostic CA projection angles were applied, including left anterior oblique (LAO) 45°, right anterior oblique (RAO) 30°, cranial (Cran) 25°, caudal (Caud) 25°, LAO 45°/Cran 25°, LAO 45°/Caud 25°, RAO 30°/Cran 25° and RAO 30°/Caud 25°. At each projection angle, 10 seconds of CA was acquired three times to obtain the average level of operator radiation.

An anthropomorphic male phantom (ART-200, RGRMS, Beijing, China) with a height of 170 cm and weight of 60 kg was used to simulate a realistic patient X-ray attenuation under a direct beam. The phantom was placed on the tabletop cushion with the left side of the chest at the angiographic C-arm’s isocenter. The maximum distance between the X-ray tube and the flat-panel detector was 150 cm. However, the distance between the flat-panel detector and the phantom was kept as close as possible during the acquisitions based on the “as low as reasonably achievable” (ALARA) principle.

**Figure 1:**
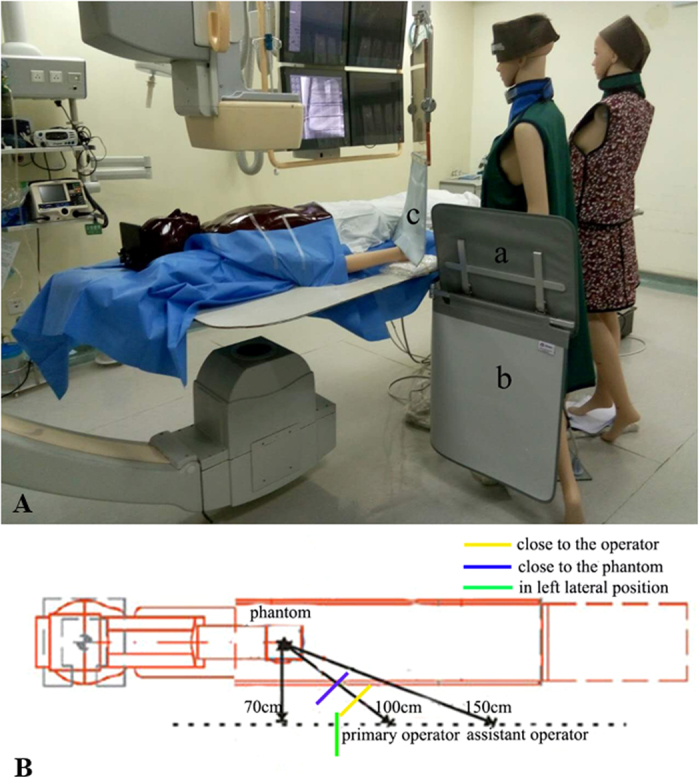
CA procedures were simulated in a cardiac catheterization laboratory using an anthropomorphic patient phantom and two manikins as the operators; these operators wore lead aprons, thyroid collars and lead caps. An accessory vertical extension (**A**), a lower body shield (**B**) and a ceiling-suspended lead screen (**C**) were used during the radiation dose rates measurements (Fig. 1A). PO and assistant operators’ standing positions in relation to the X-ray gantry and patient phantom to mimic a typical catheterization scenario using radial artery access (Fig. 1B).

At our centre, transradial catheterization was performed during CA with PCI as the preferred access route. Two 175-cm tall silicone manikins were positioned at the right side of the patient table to mimic the radial access (Fig. 1A). Typical standing positions of the PO and the assistant were assigned to the mannequins at 100 cm and 150 cm away from the X-ray tube, respectively, for the anterior-posterior projection (Fig. 1B). The perpendicular distance from the operators’ standing line to the X-ray tube was 70 cm. Based on radiation protection best practices, the operator models were dressed with lead aprons, thyroid collars and lead caps.

### Dosimeters

DoseAware real-time electronic dosimeters (Philips Medical System, Best, the Netherlands) were attached to the operators to measure the radiation dose rates at various locations. The pocket dosimeters, using solid state Si, were designed to measure personal dose equivalents in catheterization laboratories. The sensitive range of the devices is from 40 μSv/hour to 300 mSv/hour, with a 20% variation in the energy response between 33 and 100 keV^22^. A previous study^23^ reported 10–15% measurement differences between the DoseAware and TLDs in cardiology radiation fields.

The present study attached the dosimeters to the front of the operators’ lead protective aprons. We defined eight measurement points on the operators at various heights and positions close to radiation-sensitive organs, i.e., the glabella (170 cm, close to eye lenses), neck (155 cm, close to thyroid glands), left upper thorax (140 cm), epigastrium (120 cm), hypogastrium (100 cm, close to the gonads), left thigh (75 cm), left shin (40 cm) and left ankle (15 cm). The radiation dose rate (in μSv/min) at each measurement point was determined as the average readout during the three CA acquisitions from the same projection angle.

### Ceiling-suspended lead screen and shielding efficacy

A ceiling-suspended lead screen (0.5 plumbum equivalent) with an extension of flexible flaps (full dimension: 50 cm wide by 70 cm high) was investigated with regard to its placement to provide optimal radiation protection to the operators. To best represent a real clinical scenario and reflect the best practices in radiation protection, a fix mounted lower body shield (68 cm × 88 cm) and an accessory vertical extension (51 cm × 41 cm) were employed in combination with the ceiling-suspended screen during the experiment (Fig. 1A). The top of the accessory vertical extension was 30 cm higher than the patient table top. The shields were carefully placed to allow 10 cm of overlap.

First, the radiation dose rates were measured without the ceiling-suspended or lower body curtain protection to obtain baseline data. Thereafter, three ceiling-suspended lead screen placement scenarios (i.e., close to the patient phantom, in the left lateral position to the PO and close to the PO; Fig. 1B) were tested with repeated radiation dose rates measurements. When placed close to the patient phantom, the lead screen was positioned at a 45° angle and 50 cm away from the PO. In the left lateral position, the lead screen was placed close to the PO at a 90° angle. When placed close to the PO, the lead screen was kept at a 45° angle and 25 cm away from the PO. The ends of the flexible flaps of the lead screen were kept at the level of the patient surface. For all three scenarios, the lower body shield and accessory panel were mounted and kept at the same position, protecting the lower bodies of the operators on the left side and at the front of the PO (Fig. 1A, ceiling-suspended screen close to the PO).

The radiation dose rates of all measurement points on the primary and assisting operators were recorded for all three scenarios and compared with the unshielded baseline. Shielding efficacy was defined as the attenuation ratio given as a percentage (%) between the operator radiation dose rate measured using the ceiling-suspended shield and the unshielded dose rate per measurement point. All measurement results were expressed as the mean ± standard deviation (SD) and tested between the three lead screen positions and the unshielded situation using Student’s *t*-test via SPSS (Version 19.0, IBM, Armonk, NY, USA). A p-value of <0.05 was considered as significant.

## Results

**Figure 2:**
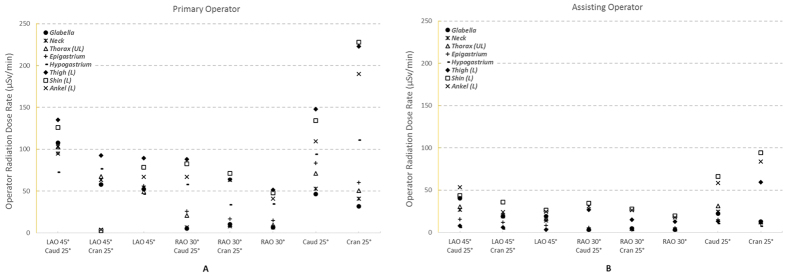
Radiation dose rate exposure to the primary (**2A**) and assistant (**2B**) operators during CA, without the table-side and ceiling-suspended lead shielding. The dose rates (μSv/min) at various operator body heights were measured on the outside of the lead aprons. LAO, left anterior oblique; RAO, right anterior oblique; Caud, caudal; Cran, cranial.

Baseline operator radiation dose rates without the ceiling-suspended and lower body shielding yielded an average of 67.2 (±49.0) μSv/min to the PO (Fig. 2A), with a minimum of 2.3 (±0.3) μSv/min at the LAO 45°/Cran 25° projection and a maximum of 227.9 (±12.5) μSv/min at the Cran 25° projection, both measured at the PO’s shin. The radiation level to the assistant was significantly lower than that to the PO at every measurement point across all CA projection angles (p < 0.003). The average was 20.1 (±19.6) μSv/min, with a minimum of 2.0 (±0.3) μSv/min at the RAO 30° projection measured at the hypogastrium and a maximum of 94.3 (±3.8) μSv/min at the Cran 25° projection measured at the shin (Fig. 2B).

Variations in radiation dose rate were observed with respect to the CA projection angle and the measurement point on the operators’ bodies. The highest radiation exposures to both operators appeared at the Cran 25°, Caud 25° and LAO 45°/Caud 25° projections, whereas the lowest was observed at the RAO 30° projection. The PO’s left shin and left thigh were exposed to the highest radiation dose rates (except at the LAO 45°/Cran 25° projection), whereas the glabella and neck were generally exposed to the lowest radiation dose rates, except at the LAO 45°/Caud 25° and LAO 45°/Cran 25° projections. Furthermore, the Cran25° projection caused the largest variation in the radiation dose rates measured at different points on the PO, whereas the LAO 45° and RAO 30° projections were associated with the smallest variations relative to the other CA projections. Similar variations were observed for the assisting operator, although with approximately half of the radiation dose rates of that to the PO. In general, the assistant’s left ankle and left shin were exposed to the highest radiation dose rates, whereas the hypogastrium was exposed to lowest dose rates. Regarding the PO, the Cran 25° projection caused the largest variation in radiation dose rates amongst the measurement points on the assistant, whereas the RAO 30° projection was associated with the smallest variation.

**Figure 3:**
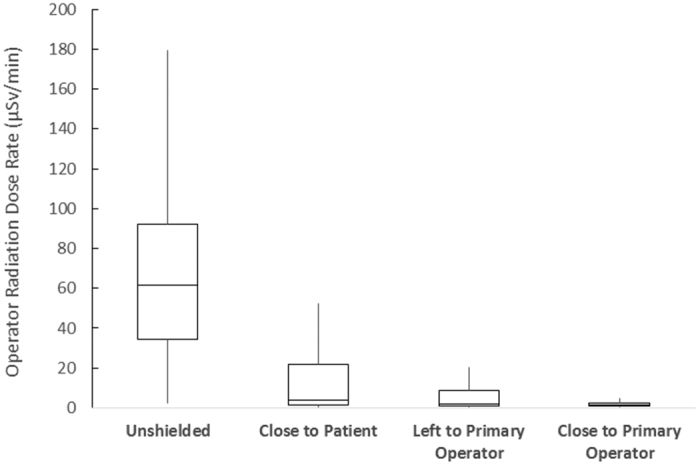
Overall ceiling-suspended lead screen shielding efficacy to the PO with respect to placement location. Boxes denote the interquartile range and median of the radiation doses rates measured at all CA angles for all body heights. The whiskers illustrate the minimum and maximum data points (excluding outliers over 1.5 box heights above the third quartile).

The use of a ceiling-suspended lead screen combined with a lower body shield significantly reduced the radiation level to the operators. The average radiation dose rates for the PO were 15.6 (±21.9) μSv/min, 12.2 (±23.0) μSv/min and 4.4 (±10.0) μSv/min when the ceiling-suspended lead screen was placed close to the patient phantom, at the left lateral position to the PO, and close to the PO. These positions resulted in 76.8%, 81.9% and 93.5% radiation reduction compared with the unshielded baseline radiation levels (Fig. 3 and Table S1). The assisting operator received average radiation dose rates of 6.0 (±5.3) μSv/min, 4.7 (±4.7) μSv/min and 2.0 (±1.5) μSv/min during the three lead screen placement scenarios, respectively, yielding shielding efficacies of 70.3%, 76.7% and 90.0%. The shielding effects of placing the ceiling-suspended lead screen close to the PO significantly differed from the other scenarios and unshielded situation (p < 0.013).

**Figure 4:**
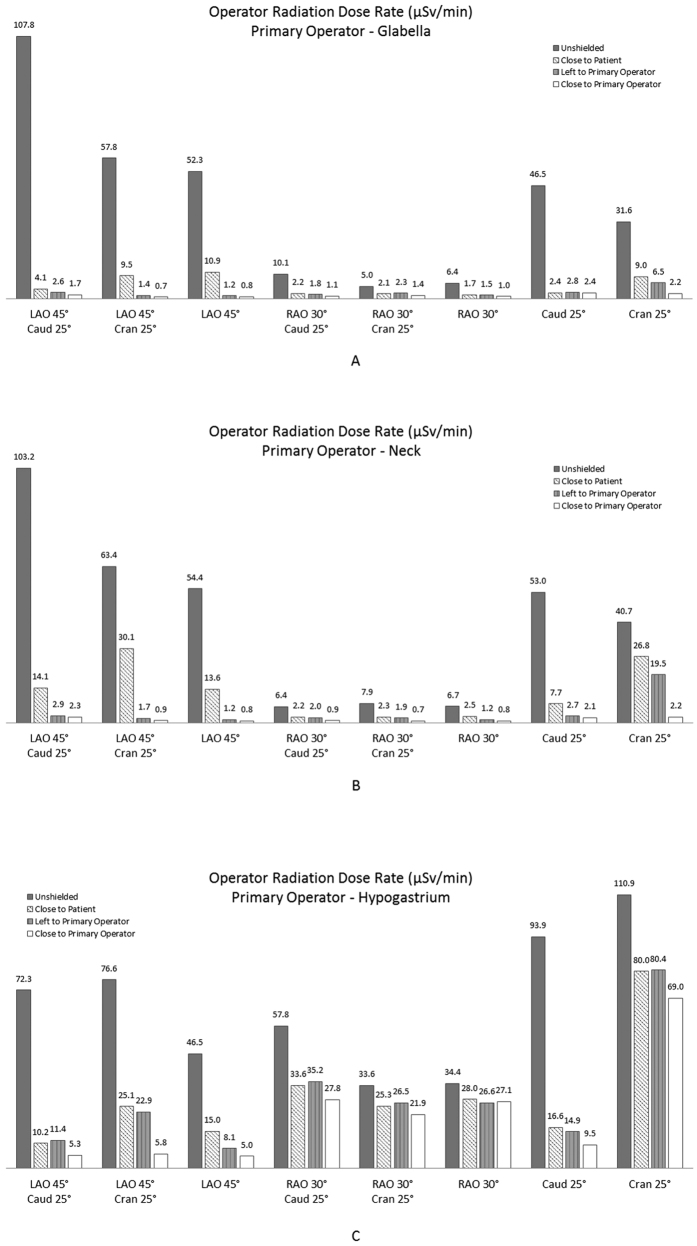
Ceiling-suspended lead-screen shielding effects for the PO in radiation sensitive areas, i.e., the glabella (**4A**), neck (**4B**), and hypogastrium (**4C**). The lead screen was placed close to the patient phantom in the left lateral position and close to the PO. LAO, left anterior oblique; RAO, right anterior oblique; Caud, caudal; Cran, cranial.

Despite receiving the highest radiation dose rates when unshielded, the lower limbs of the operators were well protected by the lower body and accessory panel, with a small variation influenced by the ceiling-suspended lead screen placement and the CA projection angle. The PO’s thigh, shin and ankle received radiation dose rates of 0.33–3.96 μSv/min after shielding was applied. The assistant’s thigh, shin and ankle received radiation at 0.67–5.37 μSv/min. The glabella and neck are close to radiation-sensitive organs. Although they received the lowest radiation dose rates when unshielded, they showed a significant reduction in dose rate when the ceiling-suspended lead screen and the table-side shields were in place. Figure 4A and B illustrate the shielding effects on the PO. Similar observations were recorded with regard to the assisting operator. Unlike the other areas of the body, the shielding effects on the hypogastrium varied largely with the CA projection angles (Fig. 4C).

## Discussion

The radiation dose rate measurements obtained in this study provide a detailed knowledge of the spatial distribution of the radiation exposure due to varying CA projection angles and measurement points on the operator’s body. Interventional cardiologists should be aware of the large differences in projection angles to improve occupational radiation safety. When clinically feasible, avoiding or substituting highly irradiating projections with less irradiating ones and consciously limiting the acquisition time of highly irradiating projections is advised, particularly during complex PCIs. For both the primary and assisting operators, a CA at the RAO 30° projection was associated with the lowest radiation rates of the routine projections angles, and this result corroborates previously published findings^7^,^8^,^24^. Kuon *et al*. revealed that operator radiation is most intense in the direction back towards the x-ray tube and least intensive towards the image detector^8^. These effects result in projections with LAO rotations that cause more operator radiation. As a consequence of the X-ray tube being under the operating table in most of the CA projections, the operators’ lower extremities are exposed to higher radiation doses than their mid and upper bodies. In addition, the left leg, which was closer to the X-ray tube, received more radiation than the right leg^20^. Despite the higher radiation level, the present study and other publication have demonstrated excellent protection of the lower extremities when table-side mounted shields are used.

The annual dose limits for the occupational exposure issued by the International Commission on Radiological Protection are 20 mSv effective dose, 20 mSv equivalent dose to lens of the eye and 500 mSv equivalent dose to the extremities^25^. Exceeding these limits raises the risk of occupational injury due to ionizing radiation. The current study showed that unshielded operator radiation easily reaches these dose limits for POs with high annual procedural volume. Therefore, it is crucial to adopt the best shielding strategy to minimize the risk of occupational radiation injury.

Although certain CA projection angles might allow substantial radiation reduction by placing the lead screen close to the patient or at the left side of the PO. However, such positions clearly provide poor shielding to the operators at other projections (e.g., the LAO 45°/Cran 25° and Cran 25° projections). Of the three scenarios tested, placing the ceiling-suspended lead screen close to the PO resulted in the best overall shielding efficacy. Up to 98.8% and 98.6% of the operator radiation rates were prevented to the PO’s glabella and neck, respectively. This shield significantly reduces the radiation hazard to the eye lens and thyroid. Although most of the measured body heights demonstrated satisfying protection efficacy, the radiation rates affecting the operator’s hypogastrium remained relatively high when associated with the cran 25° and RAO 30° projections. Awareness should be raised, and reliable lead protection should be worn around the hypogastrium.

Note that because of the greater distance to the X-ray tube and the shielding effect of the PO, the assistant is exposed to considerably lower radiation rates. Hence, when using protective devices, the placement relative to the PO is of greatest concern. Nevertheless, we found that the assistant also received the maximum protection while employing the close to PO position of the ceiling-suspended screen.

Using real-time dosimetry designed for immediate personal radiation feedback, the current study reported the operator radiation dose rate (in Sv/min). Compared with conventional dosimetry methods that require a unit conversion between the dose area product (in Gy·cm^2^) and accumulative dose measurements (in Gy) to the operator radiation dose, this device offers instantaneous staff dose display in catheterization labs. Because of variations in procedural complexity, the operator radiation dose during each procedure might be difficult to estimate. However, real-time dosimeters can effectively provide personal radiation dose monitoring during CA.

### Study limitations

The present study sought to obtain a comprehensive understanding of the radiation experienced by operators and the shielding effects in the dynamic environment of CA. Nevertheless, we recognize several limitations in the experimental protocol, including the fixed standing positions of the primary and assisting operators. In clinical practice, operators move towards or away from the radiation source based on the vascular access route applied and the X-ray gantry rotation. However, as long as the ceiling-suspended lead shield is carefully kept close to the PO towards the source of the radiation, then the influence of the radiation due to a change in position can be ignored because substantial protection is expected. We applied an anthropomorphic phantom as a mock patient to obtain a static baseline. Patient body size is known to influence radiation dose and might change the spatial profile of the operator radiation. Additional investigation during clinical procedures might be worthwhile to gain a better understanding.

## Conclusions

Effective radiation protection during CA can be achieved via the thoughtful placement of radiation shields. Given the variations in operator radiation caused by changing angiographic projection angles, ceiling-suspended lead screens should be positioned close to the PO towards the radiation source to achieve optimal shielding. Real-time dosimetry is a convenient way to monitor operator radiation via instantaneous feedback.

## Additional Information

**How to cite this article**: Jia, Q. *et al*. Operator Radiation and the Efficacy of Ceiling-Suspended Lead Screen Shielding during Coronary Angiography: An Anthropomorphic Phantom Study Using Real-Time Dosimeters. *Sci. Rep.* **7**, 42077; doi: 10.1038/srep42077 (2017).

**Publisher's note:** Springer Nature remains neutral with regard to jurisdictional claims in published maps and institutional affiliations.

## Supplementary Material

Supplementary Table S1
