# Supplementary material for: Operator Radiation and the Efficacy of Ceiling-Suspended Lead Screen Shielding during Coronary Angiography: An Anthropomorphic Phantom Study Using Real-Time Dosimeters
Source: Sci Rep. 2017 Feb 7;7:42077. doi: 10.1038/srep42077 (PMC5294580; doi:10.1038/srep42077)
Supplement: Supplementary Table S1 [file srep42077-s1.doc]

**Operator Radiation and the Efficacy of Ceiling-Suspended Lead Screen Shielding during Coronary Angiography: An Anthropomorphic Phantom Study Using Real-Time Dosimeters**

**Author list:**

Qianjun Jia¹*, MD; Ziman Chen¹*, MD; Xianxian Jiang², MSc; Zhenjun Zhao^3^, MD; Meiping Huang¹, MD; Jiahua Li¹, MD; Jian Zhuang^4^, MD, PhD; Xiaoqing Liu^5^, MD, PhD; Tianyu Hu¹, MM; and Wensheng Liang¹, MM

**Table S1. Operator radiation dose rate (µSv/min, mean±standard deviation) to the primary operator (PO) and the shielding efficacy (%) of a ceiling-suspended lead screen during coronary angiography**

| **Measurement points** | **Ceiling-Suspended Lead Screen Placement** | **LAO 45°/Caud 25°** | **LAO 45°/Cran 25°** | **LAO 45°** | **RAO 30°/Caud 25°** | **RAO 30°/Cran 25°** | **RAO 30°** | **Caud 25°** | **Cran 25°** |
| --- | --- | --- | --- | --- | --- | --- | --- | --- | --- |
| **Glabella** | Unshielded | 107.8±5.6 | 57.8±1.9 | 52.3±3.1 | 5.0±0.4 | 10.1±0.9 | 6.4±0.5 | 46.5±4.2 | 31.6±2.2 |
|  | Close to PO | 1.7±0.3 (98.5) | 0.7±0.1 (98.8) | 0.8±0.2 (98.5) | 1.4±0.3 (72.2) | 1.1±0.3 (89.1) | 1.0±0.3 (84.5) | 2.4±0.4 (94.9) | 2.2±0.3 (93.0) |
|  | Close to patient | 4.1±0.4 (96.2) | 9.5±0.7 (83.7) | 10.9±0.9 (79.1) | 2.1±0.2 (59.2) | 2.2±0.3 (78.4) | 1.7±0.3 (74.2) | 2.4±0.3 (94.9) | 9.0±0.8 (71.4) |
|  | Left lateral to PO | 2.6±0.5 (97.6) | 1.4±0.3 (97.6) | 1.2±0.3 (97.7) | 2.3±0.3 (54.5) | 1.8±0.3 (82.6) | 1.5±0.3 (76.4) | 2.8±0.4 (94.0) | 6.5±0.4 (79.3) |
| **Neck** | Unshielded | 103.2±6.8 | 63.4±3.8 | 54.4±3.7 | 6.4±0.7 | 7.9±0.7 | 6.7±0.6 | 53.0±2.9 | 40.7±2.0 |
|  | Close to PO | 2.3±0.3 (97.8) | 0.9±0.3 (98.6) | 0.8±0.2 (98.5) | 0.9±0.2 (86.0) | 0.7±0.2 (90.9) | 0.8±0.2 (88.1) | 2.1±0.3 (96.0) | 2.2±0.4 (94.5) |
|  | Close to patient | 14.1±1.5 (86.3) | 30.1±1.5 (52.6) | 13.6±1.0 (75.1) | 2.2±0.2 (65.2) | 2.3±0.4 (70.6) | 2.5±0.4 (61.8) | 7.7±0.8 (85.5) | 26.8±2.2 (34.2) |
|  | Left lateral to PO | 2.9±0.4 (97.2) | 1.7±0.3 (97.3) | 1.2±0.3 (97.8) | 2.0±0.3 (68.9) | 1.9±0.3 (76.3) | 1.2±0.3 (81.5) | 2.7±0.4 (94.9) | 19.5±1.4 (52.2) |
| **Upper left thorax** | Unshielded | 102.8±4.5 | 67.5±1.9 | 49.0±1.9 | 20.8±1.1 | 9.8±0.9 | 9.2±0.6 | 71.2±2.9 | 50.6±3.3 |
|  | Close to PO | 3.1±0.4 (97.0) | 0.8±0.2 (98.8) | 1.0±0.2 (97.9) | 0.8±0.2 (96.2) | 0.6±0.2 (93.8) | 0.6±0.3 (93.7) | 2.2±0.4 (96.9) | 2.1±0.4 (95.9) |
|  | Close to patient | 36.2±3.6 (64.8) | 42.2±2.7 (37.5) | 31.7±1.6 (35.3) | 11.9±0.7 (42.7) | 3.9±0.4 (60.2) | 3.4±0.4 (63.0) | 20.5±1.5 (71.2) | 36.0±1.6 (28.9) |
|  | Left lateral to PO | 2.6±0.5 (97.5) | 1.1±0.3 (98.4) | 0.8±0.2 (98.3) | 0.9±0.1 (95.8) | 0.7±0.2 (92.7) | 0.8±0.2 (91.3) | 1.5±0.2 (98.0) | 7.8±2.7 (84.5) |
| **Epigastrium** | Unshielded | 95.3±4.4 | 92.4±4.1 | 56.0±3.0 | 25.8±1.2 | 16.7±1.3 | 14.7±0.8 | 83.7±3.8 | 60.2±3.4 |
|  | Close to PO | 2.7±0.27 (97.2) | 1.4±0.3 (98.4) | 1.7±0.3 (96.9) | 2.3±0.2 (91.2) | 9.8±0.7 (41.5) | 6.2±0.3 (57.6) | 3.8±0.5 (95.5) | 16.7±1.1 (72.3) |
|  | Close to patient | 80.8±4.4 (15.2) | 92.3±2.5 (0.1) | 66.6±3.4 (-19.1) | 20.0±1.3 (22.5) | 16.8±1.3 (-0.4) | 14.9±1.2 (-1.4) | 52.4±2.4 (37.4) | 63.4±3.3 (-5.3) |
|  | Left lateral to PO | 92.0±4.1 (3.5) | 88.3±4.1 (4.5) | 61.4±3.1 (-9.7) | 30.0±2.4 (-16.4) | 18.0±1.0 (-7.8) | 16.6±1.6 (-13.3) | 77.6±3.4 (7.3) | 67.0±3.7 (-11.4) |
| **Hypogastrium** | Unshielded | 72.3±2.5 | 76.6±3.6 | 46.5±1.6 | 57.8±2.3 | 33.6±2.7 | 34.4±2.2 | 93.9±3.1 | 110.9±4.9 |
|  | Close to PO | 5.3±0.7 (92.6) | 5.8±0.7 (92.4) | 5.0±0.4 (89.3) | 27.8±1.6 (51.8) | 21.9±1.1 (34.7) | 27.1±2.2 (21.3) | 9.5±0.8 (89.9) | 69.0±3.1 (37.8) |
|  | Close to patient | 10.2±1.1 (86.0) | 25.1±1.3 (67.2) | 15.0±0.8 (67.6) | 33.6±1.9 (41.8) | 25.3±1.7 (24.7) | 28.0±1.4 (18.4) | 16.6±0.9 (82.4) | 80.0±3.5 (27.9) |
|  | Left lateral to PO | 11.4±0.5 (84.2) | 22.9±1.1 (70.1) | 8.1±0.3 (82.7) | 35.2±1.9 (39.1) | 26.5±1.1 (21.0) | 26.6±0.9 (22.6) | 14.9±0.6 (84.2) | 80.4±1.5 (27.5) |
| **Left thigh** | Unshielded | 135.3±3.8 | 92.4±2.6 | 89.5±4.9 | 88.0±3.7 | 63.7±4.1 | 51.4±3.4 | 147.9±3.1 | 222.7±4.4 |
|  | Close to PO | 1.4±0.3 (99.0) | 0.4±0.2 (99.5) | 0.8±0.19 (99.1) | 0.4±0.2 (99.5) | 0.3±0.1 (99.5) | 0.4±0.2 (99.3) | 1.5±0.2 (99.0) | 1.5±0.2 (99.3) |
|  | Close to patient | 1.6±0.3 (98.8) | 1.3±0.2 (98.6) | 1.4±0.3 (98.5) | 0.8±0.2 (99.1) | 0.8±0.2 (98.8) | 0.6±0.3 (98.8) | 1.0±0.3 (99.3) | 3.2±0.4 (98.6) |
|  | Left lateral to PO | 2.2±0.3 (98.4) | 1.2±0.3 (98.7) | 0.8±0.1 (99.1) | 1.1±0.2 (98.7) | 0.6±0.2 (99.1) | 0.4±0.1 (99.2) | 2.1±0.3 (98.6) | 2.1±0.3 (99.1) |
| **Left shin** | Unshielded | 126.0±4.8 | 2.3±0.3 | 78.3±3.7 | 82.6±4.2 | 71.1±4.1 | 47.8±3.6 | 140.0±6.5 | 227.9±12.5 |
|  | Close to PO | 1.1±0.2 (99.1) | 0.5±0.2 (77.2) | 0.6±0.3 (99.2) | 0.8±0.2 (99.0) | 0.5±0.2 (99.3) | 0.6±0.2 (98.8) | 1.8±0.3 (98.7) | 1.4±0.3 (99.4) |
|  | Close to patient | 1.1±0.3 (99.2) | 0.4±0.2 (84.1) | 0.6±0.2 (99.3) | 0.7±0.3 (99.1) | 0.6±0.2 (99.1) | 0.5±0.2 (98.9) | 0.8±0.2 (99.4) | 1.3±0.2 (99.5) |
|  | Left lateral to PO | 0.9±0.2 (99.3) | 0.4±0.2 (84.9) | 0.5±0.2 (99.4) | 0.9±0.2 (99.0) | 0.4±0.2 (99.4) | 0.4±0.1 (99.2) | 1.4±0.3 (99.0) | 1.3±0.2 (99.4) |
| **Left ankle** | Unshielded | 94.8±4.4 | 4.1±0.4 | 67.1±3.1 | 66.9±3.7 | 63.5±4.5 | 41.0±2.6 | 109.6±2.7 | 190.0±8.5 |
|  | Close to PO | 1.8±0.2 (98.1) | 0.7±0.2 (82.5) | 1.2±0.2 (98.2) | 1.9±0.2 (97.2) | 1.1±0.2 (98.2) | 1.1±0.2 (97.3) | 4.0±0.4 (96.4) | 3.5±0.3 (98.1) |
|  | Close to patient | 2.8±0.4 (97.1) | 0.9±0.2 (78.0) | 1.3±0.3 (98.1) | 1.8±0.2 (97.4) | 1.2±0.2 (98.1) | 1.2±0.2 (97.1) | 2.0±0.2 (98.2) | 3.7±0.3 (98.0) |
|  | Left lateral to PO | 2.9±0.3 (96.9) | 1.0±0.2 (76.1) | 1.1±0.2 (98.4) | 2.1±0.2 (96.9) | 1.2±0.2 (98.2) | 0.9±0.2 (97.8) | 3.2±0.3 (97.1) | 3.4±0.3 (98.2) |

LAO, left anterior oblique; RAO, right anterior oblique; Caud, caudal; Cran, cranial
